# A Study on the Effect of Ultrasonic Treatment on the Microstructure of Sn-30 wt.% Bi Alloy

**DOI:** 10.3390/ma11101870

**Published:** 2018-10-01

**Authors:** Shuo Wang, Jinwu Kang, Xiaopeng Zhang, Zhipeng Guo

**Affiliations:** 1 School of Materials Science and Engineering, Tsinghua University, Beijing 100084, China; saviorggg@163.com (X.Z.); zhipeng_guo@mail.tsinghua.edu.cn (Z.G.); 2 Key Laboratory for Advanced Materials Processing Technology, Ministry of Education, Beijing 100084, China

**Keywords:** ultrasonic treatment, Sn-Bi alloy, dendrite fragmentation, divorced eutectic

## Abstract

The effect of ultrasonic treatment on the microstructure of Sn-30 wt.% Bi alloy was studied at different temperatures. Results showed that the ultrasonic treatment could effectively refine the microstructure of Sn-30 wt.% Bi alloy at a temperature range between the liquidus and solidus. Application of the ultrasound could fragment the primary Sn dendrites during solidification due to a mixed effect of ultrasonic cavitation and acoustic streaming. The divorced eutectic formed when the ultrasonic treatment was applied for the whole duration of the solidification. The eutectic phase grew and surrounded the primary Sn dendrite, and pure Bi phase grew in between the Sn dendritic fragments. The mechanism of the fragmentation of dendrites and the divorced eutectic structure by ultrasonic treatment was discussed.

## 1. Introduction

Ultrasonic treatment (UST) is extensively used in purifying, degassing, and refining metallic melt. Introducing ultrasonic field in a liquid environment increases nonlinear effects such as cavitation and acoustic streaming [1,2,3,4,5]. Applying ultrasound could refine the microstructure [6,7,8,9] and improve mechanical properties [9,10,11] without using the grain refinement agent [12,13,14]. However, the implementation of ultrasonic treatment leads to direct contact between the ultrasound probe and the melt, which in turn increases the processing time [15]. The ultrasound probe must be withdrawn from the melt before complete solidification. It is relatively hard to evaluate the effective temperature range for ultrasonic treatment and effort had been made to study the UST effect at different temperatures. Liu et al. [16] investigated the effect of ultrasonic vibrations at different temperatures of the AZ91 melt on the microstructure UST introduced in the nucleated stage, which can be greatly refined and attributed to the combination of the promoted heterogeneous nucleation and dendrite multiplication. Jian et al. [17] make efforts to evaluate the effect of UST on grain refinement at various stages of solidification. They designed an intermittent treatment with an interval temperature of 5 °C and different treatment times in the mushy zone. It was found that intermittent treatment is more efficient than continuous treatment at a certain temperature in terms of grain refinement. They concluded that the dominant mechanism for grain refinement using acoustic vibrations is cavitation-induced heterogeneous nucleation. The UST equipment used in these works [16,17] was typically that with ultrasonic probe sintered into the melt. It was hard to realize a long time treatment in the mushy zone. Kang et al. [18,19] introduced and employed a so-called upward ultrasonic treatment. Accordingly, the ultrasound probe pointed upwards and the melt to be treated is weld or cast together with the probe of the same diameter.

In this paper, the upward ultrasonic treatment was adopted to investigate the effect of ultrasonic treatment on the solidification microstructure at different temperature ranges.

## 2. Materials and Methods

### 2.1. Experimental

**Figure 1:**
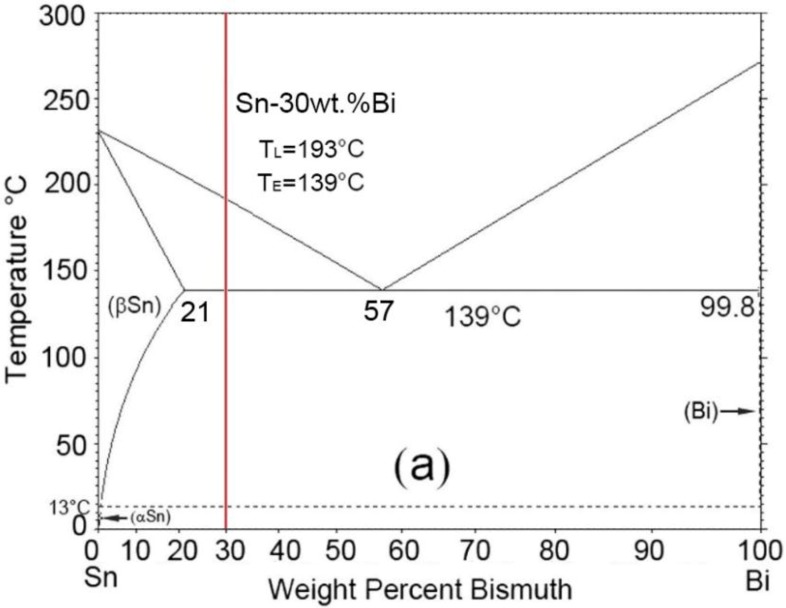
Phase diagram of the Sn-Bi alloy [20,21].

A Sn-30 wt.% Bi alloy with a wide solidification temperature range (~54 °C according to the phase diagram [20,21]) was employed in the current work. According to the phase diagram (see Figure 1), the melting point of the Sn-30 wt.% Bi is low (below 200 °C), which is very convenient for the thermal control in the experiment.

**Figure 2:**
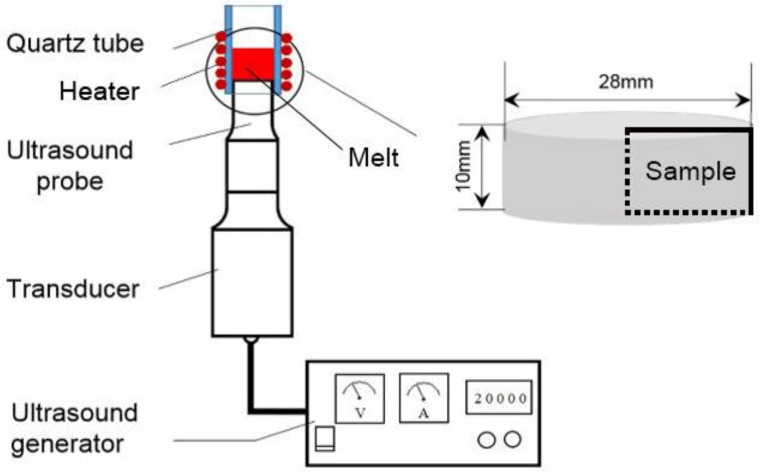
Schematic diagram of ultrasound device.

Figure 2 shows a schematic illustration of the upward ultrasonic treatment system. The ultrasonic device contained an ultrasound generator and an output terminal with continuously adjustable power in a range of 0–600 W. The vibration frequency was 20 kHz and the diameter of the probe was 26 mm [19]. A quartz tube with an inner diameter of 28 mm and a length of 40 mm was put on top of the probe. Fire clay was used to seal the gap between the tube and the side of the probe. The seal avoided direct contact between the tube and the probe, thus preventing possible breaking of the tube during ultrasonic treatment. Heating coils were used to melt the Sn-Bi specimen attached to the probe tip. A controller was applied to control the heating rate and the melt temperature. The ultrasound was generated from the bottom and directed inside the melt through the specimen.

The alloy was made by melting pure Sn and pure Bi according to the pre-set weight ratio in a graphite crucible with the protection gas of Argon. The alloy was held at 300 °C for one hour for homogenization, and then cast into a quartz tube affixed to the probe tip.

During the experiment the Sn-Bi specimen attached to the probe tip was heated up to 220 °C by the electrical resistance coils. After melted, the specimen was held for 30 min at 220 °C and then cooled. The ultrasonic device was then turned on to introduce the ultrasound in the melt. Three temperature conditions were considered, including (1) above the liquidus; (2) in-between liquidus and solidus, and (3) the whole duration of solidification. Table 1 shows the parameters for ultrasonic treatment. The solidified samples were cut off the probe tip and then sectioned into specimens for further microstructure observation using a Keyence VHX-6000 Digital Microscope (Osaka, Japan).

### 2.2. Modeling

#### 2.2.1. Pressure Distribution in Melt

The dendritic fragmentation in an ultrasound field was closely related to the ultrasonic pressure and the interaction with the oscillating bubbles [22,23]. The pressure distribution in the melt under experimental conditions was simulated by solving the Helmholtz equation (Equation (2)) for acoustic pressure using a software named COMSOL Multiphysics (COMSOL, Inc., Burlington, MA, USA) [22,24]. Table 2 listed the related properties and parameters of the alloy used in the experiment [24,25,26,27,28].

(1) $$\frac{\kappa P_{a}}{\rho_{0}}+\nabla \cdot \left(\frac{1}{\rho_{0}}\nabla P_{a}\right)=0$$

where $\rho_{0}$ is the liquid density, $\kappa =\omega /c_{0}$ is the wave number, and *ω* = 2π*f* is the angular frequency, *f* is the frequency of the sound wave, and *c*_0_ is the speed of sound in the liquid. The boundary conditions applied were:

(1) Pressure source $P_{a}=P_{A}\cos(\omega t)$, at the surface of the probe tip, where $P_{A}=\sqrt{2\rho_{0}c_{0}W/A}$ *W* is the ultrasound power, and *A* is the area of probe tip;
(2) Sound hard boundary condition with zero normal derivative of the pressure $\partial P_{a}/\partial n=0$ at the sides of the probe;
(3) Sound soft boundary condition at the top end of domain to simulate the liquid/air interface (*P_a_* = 0);
(4) Impedance, *Z* = *ρ_q_* · *c_q_* at liquid/glass interface.

#### 2.2.2. Pressure Amplitude of an Oscillating Ultrasound Bubble

The Gilmore model [29] was used to calculate the pressure evolution of an oscillating ultrasound bubble according to Equations (2)–(5) using COMSOL Multiphysics (COMSOL, Inc., Burlington, MA, USA) [22,24].

(2) $$\left(1-\frac{\dot{R}}{C}\right)\dot{R}\ddot{R}+\frac{3}{2}{\dot{R}}^{2}\left(1-\frac{\dot{R}}{3C}\right)=\left(1+\frac{\dot{R}}{C}\right)H+\frac{R\dot{R}}{C}\left(1-\frac{\dot{R}}{C}\right)\frac{dH}{dR}$$

(3) $$C=\frac{n(P+B)}{\rho }{\left(\frac{P+B}{P_{0}+B}\right)}^{\frac{n-1}{2n}}$$

(4) $$H=\frac{n}{n-1}\frac{{\left(P_{0}+B\right)}^{\frac{1}{n}}}{\rho }\left[{\left(P+B\right)}^{\frac{n-1}{n}}-{\left(P_{0}+B\right)}^{\frac{n-1}{n}}\right]$$

(5) $$P(R)=\left(P_{0}+\frac{2\sigma }{R_{0}}\right){\left(\frac{R_{0}}{R}\right)}^{3\gamma }-\frac{2\sigma }{R}-\frac{4\mu \dot{R}}{R}$$

where *R*, *P*, *H* and *C* is the radius, pressure at the bubble wall, enthalpy and local speed of sound in the liquid, respectively; *R*_0_ is the initial bubble radius, *P*_0_ is the liquid ambient pressure at 1 atm and *P*_∞_ = *P*_0_ + *P_a_* is the pressure at infinite distance from the bubble.

## 3. Results

### 3.1. Microstructure

**Figure 3:**
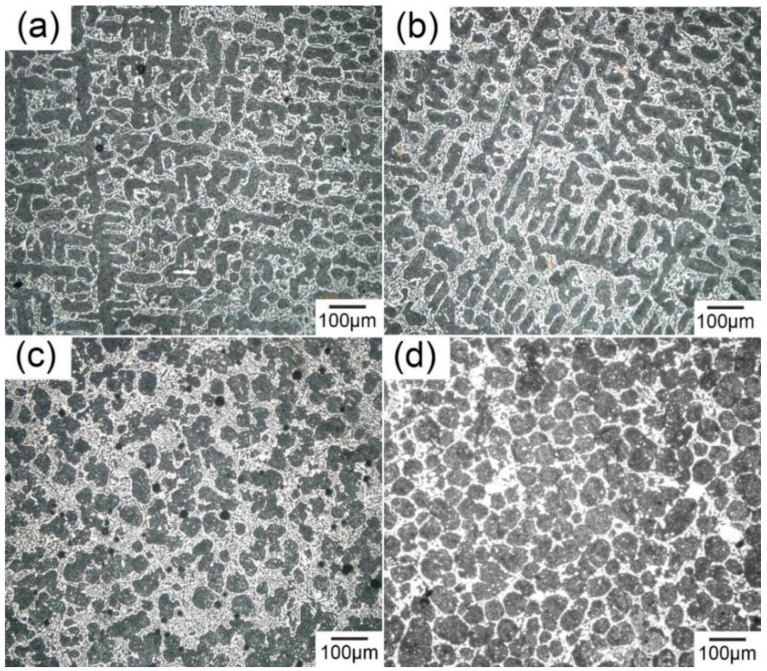
The microstructure of Sn-30 wt.% Bi alloy near probe (**a**) without UST and (**b**) with UST at 200 °C for 120 s; (**c**) with UST from 180 °C to 150 °C; and (**d**) with UST from pouring to totally solidified.

Figure 3 shows the microstructure of the Sn-30 wt.% Bi alloy without and with UST. Figure 3a shows that without ultrasonic treatment, the microstructure exhibited typical dendrites of *β*-Sn (dark) and eutectic structure (gray). The average size of the *β*-Sn dendrite in Figure 3a,b was 500–1000 μm, and the secondary arm spacing was ~50 μm. Figure 3b shows that, with an ultrasonic treatment at 200 °C for 120 s, the microstructure was similar to that without UST. However, the microstructure changed significantly when the ultrasonic treatment was introduced at 150–180 °C. As shown in Figure 3c, the *β*-Sn dendrites changed into small spheres of ~45 μm, indicating that the effective UST temperature was within the solidification freezing range. In addition, Figure 3c shows that cavitation bubbles of 20–50 μm were trapped in the solidification structure. When ultrasonic treatment lasted for the whole duration of solidification, no *β*-Sn dendrites or eutectic exhibited. Figure 3d shows that only small *β*-Sn spheres of ~50 μm as well as Bi phase (white) could be observed. Both Sn spheres and Bi phases were larger than these via UST at 180–150 °C. The ultrasonic treatment modified both primary Sn and the eutectic phase.

### 3.2. The Cavitation Bubble

**Figure 4:**
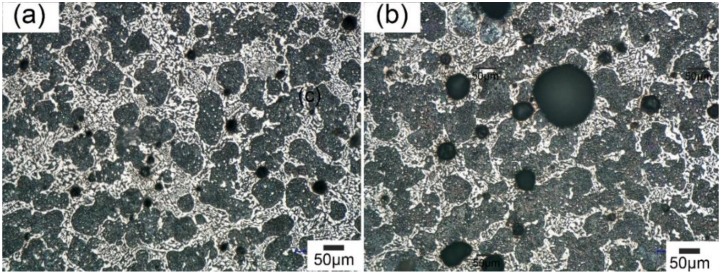
The cavitation bubbles in solidified Sn-30 wt.% Bi alloy with UST (**a**) 2 mm away from the probe; (**b**) 7 mm away from the probe.

The oscillation of the cavitation bubble was one of the driving forces for grain fragmentation during ultrasonic treatment [23,30,31,32]. As shown in Figure 4a,b, cavitation bubbles exhibited in the microstructure when UST was introduced. The cavitation bubbles were mainly ~20 μm located close to the probe while they increased to ~50 μm at 7 mm away from the probe.

**Figure 5:**
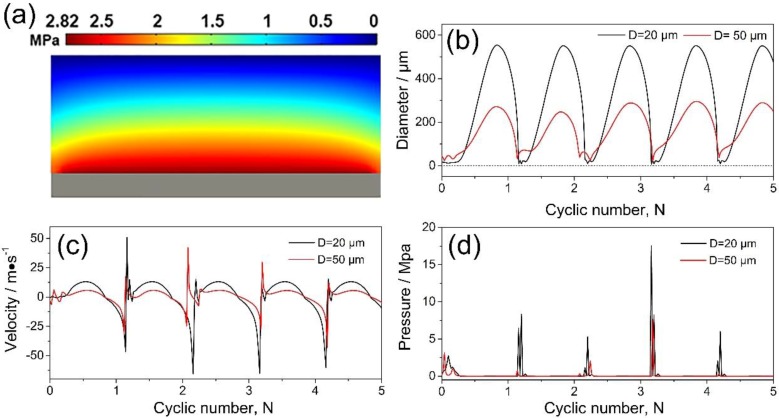
Simulation results for UST of Sn-30 wt.% Bi melt (**a**) instantaneous pressure distribution in the melt (**b**) evolution of the bubble diameter; (**c**,**d**) velocity and pressure at the bubble wall when subjected to alternating acoustic pressure. For the two cases, the initial diameter of the cavitation bubble was 20 μm and 50 μm respectively.

Using a similar method as that in References [22,24,32], Figure 5a shows the instantaneous pressure distribution in Sn-30 wt.% Bi melt subjected to an ultrasound of *f* = 20 kHz at time t = 0 s. An acoustic pressure of ~2.82 MPa was generated at the probe tip. To simulate the cavitation bubble, the Gilmore model was adopted [29] and the pressure for locations 2 mm and 7 mm away from the probe was set as 2 MPa and 0.5 MPa respectively. Figure 5b,c show the diameter and velocity at the bubble wall with an initial diameter of 20 μm and 50 μm, respectively. The velocities at bubble wall reached nearly ±50 m/s and ±30 m/s for cavitation bubbles with an initial diameter of 20 μm and 50 μm respectively. The pressure was calculated according to Equation (5) and as shown in Figure 5d, the maximum pressure at the bubble wall was ~18 MPa.

## 4. Discussion

### 4.1. The Effect of UST on the Eutectic

**Figure 6:**
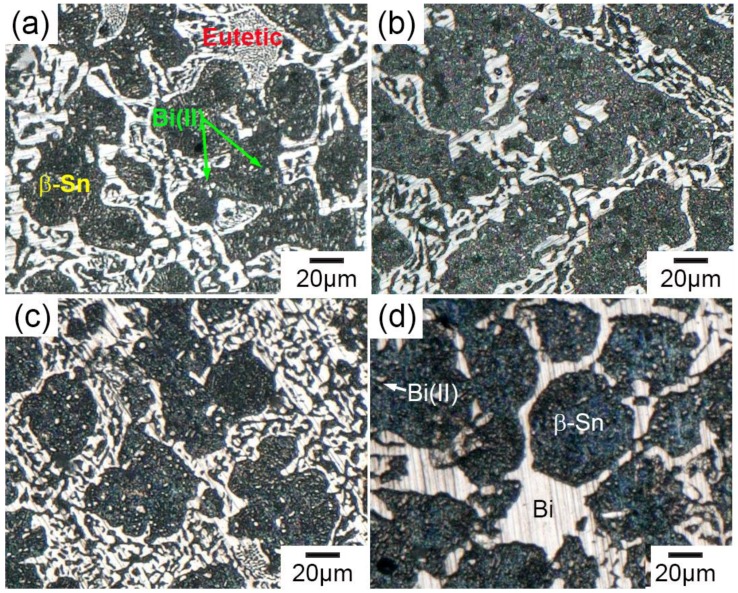
Micrographs of the microstructure of Sn-30 wt.% Bi alloy (**a**) without and (**b**) with UST at 200 °C for 120 s; (**c**) with UST from 180 °C to 150 °C; and (**d**) with UST from melt pouring to totally solidified.

Figure 6 shows the microstructure of Sn-30 wt.% Bi alloy under different UST parameters. The equilibrium solidification structure of Sn-30 wt.% Bi comprised primary *β*-Sn, eutectic (Sn and Bi), and secondary Bi precipitate. The eutectic structure exhibited a typical lamellar morphology, and distributed at the grain boundary of the primary *β*-Sn. UST treatment from 180 °C to 150 °C changed the morphology of the primary *β*-Sn phase and UST treatment during the whole solidification modified the morphologies of both the primary Sn and eutectics. The volume fraction of these phases was calculated based on the microstructure in Figure 3, and compared to that calculated using the Sn-Bi phase diagram, as listed in Table 3.

Based on the lever rule of the phase diagram, the volume fraction of the primary *β*-Sn was 75% and 25% for the eutectic. For specimens #1–3, the primary *β*-Sn phase occupied 60% whereas the Bi precipitate only occupied 3%. UST at 180–150 °C modified the morphology of primary Sn phase but exhibited little influence on the volume fraction of each phase. On the other hand, applying UST during the whole solidification increased the primary *β*-Sn phase by 70% with an additional 8.2% Sn. Comparing with sample #3, the eutectic Sn phase also decreased from 13% to 3.3%. Both the fraction of Sn and Bi indicated that the divorced eutectic occurred when UST lasted for the whole solidification.

**Figure 7:**
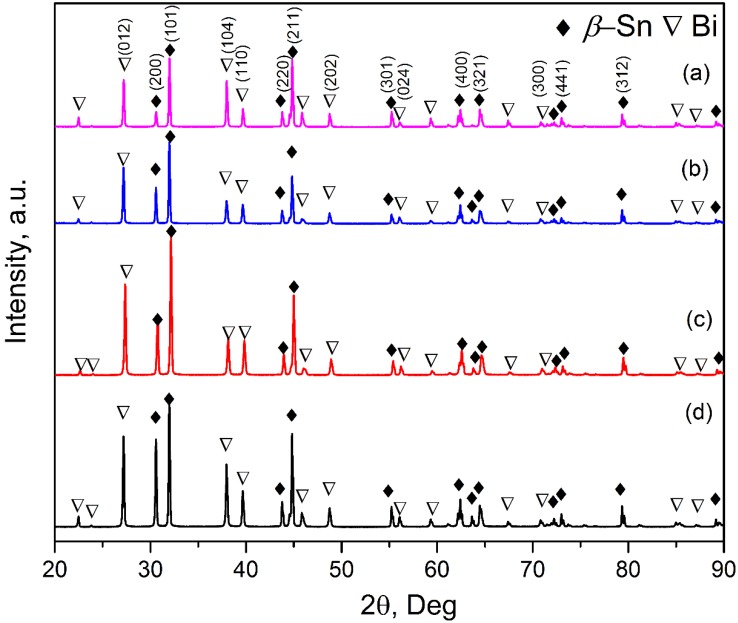
XRD patterns of the Sn-30 wt.% Bi alloy (**a**) without and (**b**) with UST at 200 °C for 120 s; (**c**) with UST from 180 °C to 150 °C; and (**d**) with UST from melting pouring to complete solidification.

Figure 7 shows the XRD patterns of the alloy without and with UST. The peaks of *β*-Sn and Bi phases exhibited and no clear difference of the peak intensity of *β*-Sn and Bi phases could be observed except that the intensity weakened for Bi (104). The intensity of typical *β*-Sn and Bi phases increased when UST was applied, including *β*-Sn (200), *β*-Sn (101), *β*-Sn (211), *β*-Sn (301), Bi (012), Bi (104), Bi (110) and Bi (202). Similar phenomena were also observed in [33] for the Sn-62 wt.% Bi. The *β*-Sn was significantly refined for samples #3–#4 when UST was introduced. Divorced eutectic occurred when UST was introduced, leading to an increase of separated *β*-Sn and Bi phases.

### 4.2. The Fragmentation Mechanism of UST

**Figure 8:**
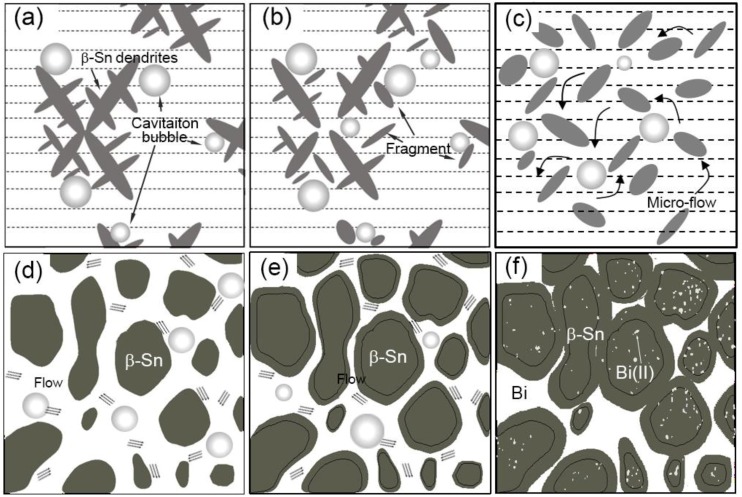
(**a**–**c**) The fragmentation mechanism of the cavitation bubbles on the dendrites; and (**d**–**f**) the formation of divorced eutectic structure when ultrasonic treatment was applied.

Figure 8a–c shows a schematic illustration of the fragmentation mechanism for the UST. The primary *β*-Sn dendrites nucleated when temperature dropped below the liquidus. When ultrasound was introduced, the cavitation bubbles were generated and oscillated with high frequency. The oscillation of the cavitation bubbles fragmented dendrites into smaller grains which were then moved by the acoustic streaming. These fragments would serve as nucleation sites for new *β*-Sn phase. A possible mechanism for dendritic fragmentation was local remelting induced by the dissipation of energy during the oscillation of cavitation bubbles [34]. But when UST was introduced above the liquidus, no dendrites existed. Although small solidified structure formed due to the chilling of the probe, they would melt immediately due to the surrounding high temperature. Accordingly, an effective UST temperature should be within the solidification freezing range.

As shown in Figure 8d,e, the Sn phase of the eutectic preferred to grow on the surface of the primary *β*-Sn phase because less nucleation energy was required. The volume fraction of the primary *β*-Sn phase was 60%, or in other words, large surface area was exposed to the melt and the required migration distance for Sn atoms became short. At high solid fraction, intense melt flow were induced by the nonuniformity of acoustic pressure [35] around the primary *β*-Sn spheres, facilitating the dissipation of the rejected Bi atoms. Accordingly, Sn tended to grow on the surface of existing *β*-Sn phase, and Bi phase formed in-between the Sn spheres, leading to the formation of the divorced eutectics (see Figure 8f).

## 5. Conclusions

The effect of ultrasonic treatment on Sn-30 wt.% Bi alloy was investigated using the probe pointing upward method. The main conclusions are:

(1) The primary *β*-Sn dendrites were fragmented into smaller grains of ~40 μm when the temperature of the ultrasonic treatment was in between liquidus and solidus of the alloy. Divorced eutectic exhibited when the treatment lasted for the whole duration of the solidification.
(2) The UST effective temperature range is between the liquidus and solidus. However, there is no effect when UST is introduced above the liquidus.
(3) The oscillation of the cavitation bubbles broke the solidified Sn dendrites into small fragments which further served as nucleation sites. The ultrasonic treatment generated micro flow around the primary Sn spheres when high solid fraction existed at eutectic temperature, facilitating the dissipation of the rejected Bi atoms, and resulting in the divorced eutectics with the separated growth of *β*-Sn and Bi phases.

## Figures and Tables

**Table 1:** The Ultrasonic treatment conditions for the Sn-30 wt.% Bi specimen.

No.	UST Temperature Range	UST Power (W)
1	-	-
2	220 °C to the end of Solidification	120
3	200 °C for 120 s	120
4	180 °C to 150 °C	120

**Table 2:** Physical properties of the Sn-30 wt.% Bi [22,24,25,26,27,28].

Parameters	Symbol/Unit	Value
Melting point	T_M_/°C	193
Density of liquid alloy	*ρ*_0_/kg·m^−3^	7.89 × 10^3^
Sound speed in liquid alloy at T_M_	*c*_0_/m·s^−1^	2464
Viscosity of liquid	*μ*/Pa·s	1.45 × 10^−3^
Surface tension	*σ*/N·m^−1^	0.388
Density of quartz	*ρ_q_*/kg·m^−3^	1400
Sound speed in quartz	*c_q_*/m·s^−1^	2380 *
Ultrasound power	W/W	120
Probe diameter	d/m	2.6 × 10^−2^
Frequency	f/Hz	20,000
Gas polytrophic exponent	*γ*	1.4
Empirical constant	*B*/atm	3046 *
Empirical constant	*n*	7.025 *

* Due to lack of data, the speed of sound in pure tin was assumed for the alloy, the values of empirical constants *B* and *n* for water were used and speed of sound in glass was used.

**Table 3:** Volume fraction of various phases for the Sn-30 wt.% Bi specimen.

Specimen	From Primary *β*-Sn/%			Eutectic/%			Total/%	
	Sn	Bi_II_	Subtotal	Sn_E_	Bi_E_	Subtotal	Sn	Bi
Phase diagram	59.2	15.8	75	10.8	14.2	25	70	30
No. 1	59.0	2.7	61.7	10.5	27.8	38.3	69.5	30.5
No. 2	57.7	2.6	60.3	13.5	26.2	39.7	70.2	28.8
No. 3	59.8	2.5	62.3	13.0	24.7	37.70	71.8	28.2
No. 4	68.2	2.5	70.7	3.3	26.0	29.3	71.5	28.5
